# Preparation and Performance of Resin-Gel–Rubber Expandable Lost Circulation Material Blend

**DOI:** 10.3390/gels9110862

**Published:** 2023-10-30

**Authors:** Jinzhi Zhu, Erbiao Lou, Shaojun Zhang, Haiying Lu, Ziwu Wang

**Affiliations:** PetroChina Tarim Oilfiled Company, Korla 841000, China

**Keywords:** lost circulation materials, resin–rubber blend, water swelling, plugging mechanism

## Abstract

Aiming at the complex strata, lost circulation often occurs. and lost circulation control becomes a difficult issue. A drilling fluid loss accident delays the drilling progress and even causes major economic losses. If we take a self-made sodium polyacrylate grafting and modify a starch water absorbent resin, using an amphiphilic compatibilizer as raw material through mechanical blending and chemical compatibilization, we can synthesize a resin–rubber blend swelling lost circulation material. This material presents a good resistance to anti-high-temperature performance, but the quality declines while the temperature is higher than 363 °C, and with the increasing temperature, the water-swelling expansion ratio becomes higher. The range of the water-swelling expansion ratio is 8 to 25 times and the water swelling rate becomes larger along with the reduced diameter of the lost circulation materials and decreases with the increasing salinity. The resin-rubber blend swelling lost circulation material after water swelling has excellent toughness and high elastic deformation capacity, thus, forming a 7 Mpa to 2 mm fracture via expansion, extrusion, deformation, and filling, which presents a good performance for fracture plugging and realizes the purpose of lost circulation control.

## 1. Introduction

Lost circulation accidents happen when a large amount of drilling fluid leaks into a formation under the effect of differential pressure, and they often occur while drilling in a formation with fracture development [1,2,3]. The occurrence of lost circulation delays the drilling process, increases drilling cost, and causes significant economic loss. To address the problem of lost circulation, researchers have devoted extensive efforts to the development of treatment measures. Among them, plugging with drilling (in which a lost circulation material (LCM) is injected into a formation with the drilling fluid and exerts its plugging effect via the LCM particles’ stacking and bridging, as well as interaction) has proven to be an effective lost circulation control method over the past decades [4,5,6].

Excellent LCMs for use while drilling need to have good temperature resistance, high strength, and adaptability to fractures of different widths to efficiently plug deep formations [7,8]. LCMs commonly used while drilling include bridging, high-water-loss, and resin gel LCMs [7,9,10,11,12]. For example, Amanullah et al. developed a series of granular bridging LCMs using date palm seeds, which displayed a bearing resistance capacity of >10 MPa for 2-mm fractures but average temperature resistance performance [13]. The gradation of commonly used bridging and high-water-loss LCMs is poor, resulting in their low plugging strength and, thus, failure to effectively plug large fractures in deep formations.

Superabsorbent resins are a class of water-swellable polymers with a certain degree of crosslinking and strong hydrophilic groups, such as carboxyl groups. They are insoluble in both water and organic solvents and have unique properties. They can absorb amounts of water that are hundreds or even thousands of times heavier than their own weights, and the gels formed after water absorption and swelling have enhanced water retention capacity and weather resistance [14,15,16]. Water-absorbent resins can absorb water and swell in a lost circulation formation, possess good deformability and anti-dilution properties, and can effectively plug a lost circulation formation via the dual effects of filling/plugging and squeezing/compacting. Owing to their controllable swelling ratio and high plugging strength, superabsorbent resin gels have received significant attention in the lost circulation control field in recent years [14].

Researchers have devoted extensive efforts to optimizing the performance of superabsorbent resin gel LCMs. It has been demonstrated that the introduction of additives can optimize the strength and stability of superabsorbent resin gels. To address the poor stability and low strength of water-absorbent resin gels, a water-absorbent resin LCM named PQ was developed with acrylic acid (AA) and acrylamide (AM) as the main raw materials; meanwhile, the addition of ultrafine CaCO3 effectively improved the strength of the resin gel [3]. Huang et al. prepared a composite LCM based on nano-acrylic resin and nano-silica and showed that the introduction of nano-silica improved the strength of the resin gel and the LCM effectively plugged fractures in shale formations [17]. Wang et al. introduced graphene oxide in a resin-gel system, and the functional groups on the surface of graphene oxide formed covalent and hydrogen bonds with the three-dimensional network structure of the water-absorbent resin, resulting in a new network that significantly optimized the thermal stability (>160 °C) of the resin gel [18]. In addition to additives, the introduction of a second network into a resin gel can also optimize its performance. For example, a double-network, high-temperature-resistant, superabsorbent resin-gel LCM was prepared using 2-acrylamide-2-methylpropanesulfonic acid (AMPS) to introduce sulfonic acid groups, and the strength of the LCM was maintained at 21,000 Pa after 10 h at 180 °C [9]. Lai et al. developed a water-absorbent resin-gel LCM named DNG with a “rigid-flexible” double network structure. The entanglement between the networks solved the problem of resin molecular chain breakage at high temperatures, and the network structure of this resin-gel LCM was still intact at a deformation of 95%, possessing good strength and high temperature resistance [3]. 

The development of LCMs with ultra-high water absorbency is of great significance for the realization of high-strength plugging. In this study, with a homemade sodium polyacrylate-modified starch water-absorbent resin gel, amphiphilic compatibilizer, and elastomer rubber as the main raw materials, we synthesized a resin-gel–rubber expandable LCM blend via mechanical blending and chemical compatibilization. The water absorption and swelling properties, thermal stability, salinity tolerance, and lost circulation control performance of the resin-gel–rubber expandable LCM blend were experimentally investigated, and its lost circulation control mechanism for fractures was discussed.

## 2. Results and Discussion

### 2.1. Preparation of Expandable LCM Blend

#### 2.1.1. Preparation Principle

**Figure 1:**
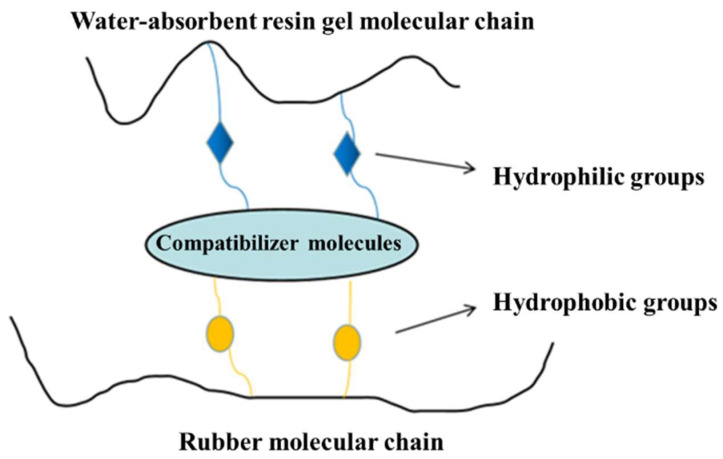
Schematic diagram of action mechanism of amphipathic compatibilizer molecules.

Resin-gel–rubber blending refers to the blending of elastomer rubber and water-absorbent resin gel under physical and chemical effects. Mechanical blending achieves the dispersion of the water-absorbent resin-gel particles in rubber via simple shearing and extruding. This can easily lead to poor compatibility between the resin and rubber, and thus, the resin gel can easily detach from the rubber after water absorption. To avoid this problem, in this study, we coupled mechanical blending and chemical compatibilization to synthesize the resin-gel–rubber expandable LCM blend. Specifically, with NBR and AA as raw materials, we prepared a compatibilizer through solution polymerization. The homemade compatibilizer contained hydrophilic groups, such as carboxyl groups, and lipophilic groups, as shown in Figure 1. The compatibilizer molecules played a “bridging” role between the water-absorbent resin gel and rubber, connecting their molecules, preventing the water-absorbent resin gel from detaching from the rubber, and improving the resin-gel–rubber expandable LCM blend’s resistance to mechanical deformation.

#### 2.1.2. Preparation of Expandable LCM Blend

In this study, we blended the water-absorbent resin gel and rubber via physical blending, resulting in the product’s simple operation, low cost, and high water absorbency.

**Figure 2:**
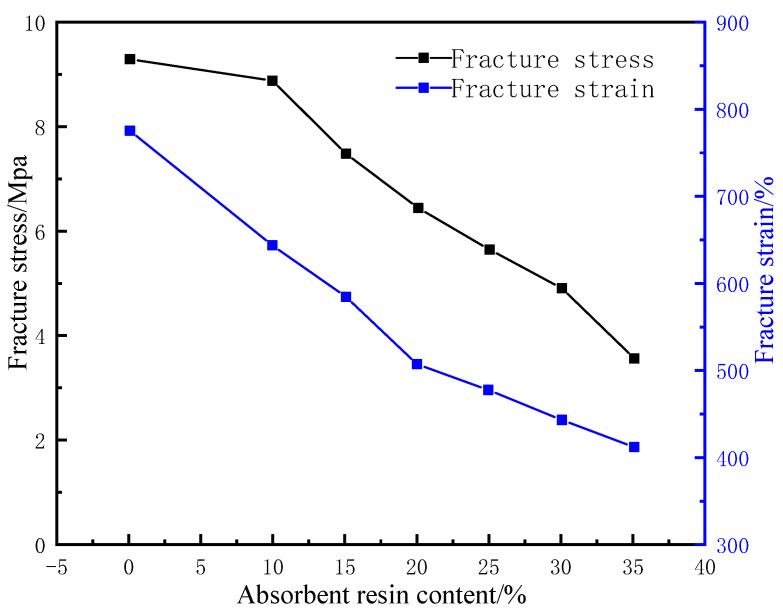
Effect of water-absorbent resin gel dosage on mechanical properties of resin-gel–rubber expandable LCM blend.

The rubber matrix and compatibilizer were mixed at a ratio of 1:0.2 and then mixed with the water-absorbent resin gel pre-moistened with deionized water at a certain mass ratio for 15 min in the closed mixer under a working temperature of 120 °C and a rotational speed of 45 r/min. With the mass of the rubber matrix as the baseline, 2% polymerized sulfur, 3% nano zinc oxide, 1% antioxidant RD, 3% stearic acid, and 3% vulcanization accelerator NA-22 were added and mixed for 10 min before discharging. The product was then pressed into a thin sheet and left to dry at room temperature for 24 h. Afterward, the sheet was placed in the flat vulcanizer for 30 min under a vulcanization pressure and temperature of 15 MPa and 120 °C, respectively. At the end of vulcanization, the pellet was taken out and kept at room temperature for 24 h. Two sample strips with a thickness of 3 mm, a length of 20 mm, and a width of 10 mm were then prepared using a CPJ-30 punching machine and placed in deionized water at 60 °C to soak for 24 h. The surface water was wiped off before testing the swelling ratio. The tensile strengths of the samples were also determined. The results of the experiments are shown in Figure 2. The samples were compared with the WCB and MMQ resin co-modified silicone molded rubber with a maximum tensile strength of 4.6 MPa prepared by Hao et al. [19]. The tensile strength of the gel–rubber expandable LCM mixture with a 25% water-absorbent resin dosage was 5.7 MPa.

**Figure 3:**
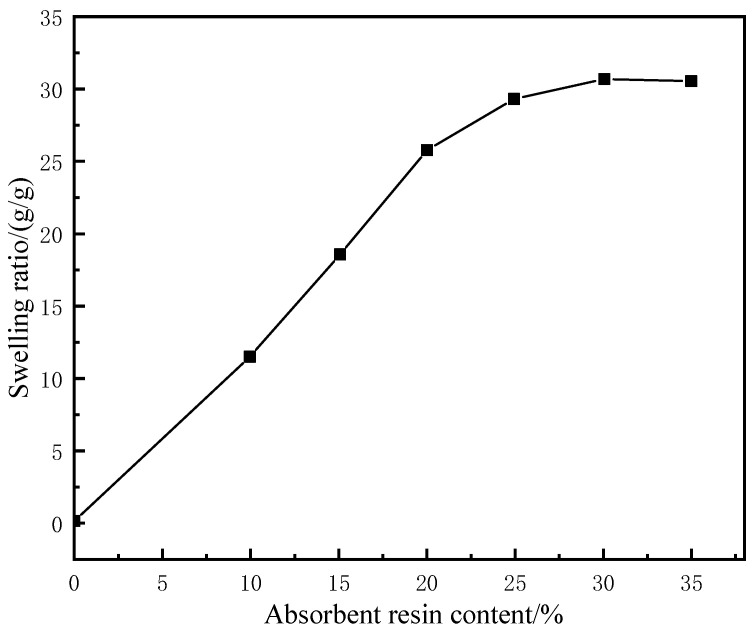
Effect of water-absorbent resin–gel dosage on the swelling ratio of resin-gel–rubber expandable LCM blend.

Under fixed concentrations of the other components, with the increase in the water-absorbent resin-gel dosage, the water absorbency of the LCM gradually increased before stabilizing, while the tensile strength and elongation at break showed significant downward trends (Figure 3). This is because the increased initial addition of water-absorbent resin gel resulted in greater water absorption and a higher swelling ratio, putting pressure on the surrounding rubber. The hydrophilic components then formed channels to the external environment, thus, precipitated under the pressure of the rubber, reaching an approximate saturation state. Meanwhile, the excess water-absorbent resin gel was unevenly distributed in the rubber matrix, which caused a stress concentration defect in the resin-gel–rubber expandable LCM blend, leading to a decrease in tensile strength and elongation at the break. To maintain the tensile strength while improving the water absorbency, the water-absorbent resin-gel dosage should be 25%.

#### 2.1.3. Compatibilizer Synthesis

Introducing a compatibilizer during the blending of the rubber and water-absorbent resin gel can increase the thickness of the interface layer between the blend components and strengthen their bonding force to a certain extent. The intermolecular bonding force promotes the combination of two incompatible polymers, thereby yielding a stable blend product, preventing the water-absorbent resin gel from detaching from the rubber and achieving better compatibility.

The NBR was weighed and dissolved in toluene in a three-necked flask via ultrasonication for 6 h at a concentration of 10%. Then, AA and azobisisobutyronitrile (AIBN) were added to the solution, and the flask was sealed and heated at 60 °C for 4 h. The gel was dried in a drying oven at 60 °C and then soaked in distilled water for 48 h. A Soxhlet extractor was used to purify the polymer product, which was then dried again. The dried rubber block was pulverized and extracted with boiling acetone for 36 h using the Soxhlet extractor to remove free rubber. The insoluble material was dried under a vacuum pressure of 100 psi, and the product with a constant mass was the compatibilizer.

The grafting rate and grafting efficiency represent the degree and rate of reaction involved in the polymerization reaction to form the target copolymer, and they can be effectively used to evaluate the reaction degree and rate of monomers:

Grafting rate = Mass of grafted monomers/Mass of grafted copolymers × 100% = (C_0_ − C_1_)/C_2_ × 100%, (1)

Grafting rate = Mass of grafted monomers/Mass of grafted copolymers × 100% = (C_0_ − C_1_)/C_2_ × 100%, (2)

where C_0_ is the added mass of monomer AA, C_1_ is the total mass of the dried product after the first extraction with distilled water, and C_2_ is the total mass of the dried product after the second extraction with acetone.

The following table shows the influence of the initiator and grafting monomer on the compatibilizer’s grafting rate and efficiency.

**Figure 4:**
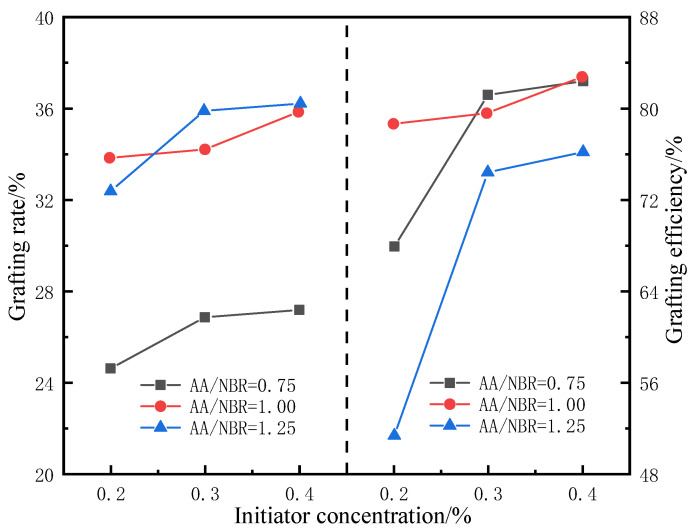
Influence of initiator and grafting monomer on compatibilizer’s grafting rate and efficiency.

As shown in Figure 4, at a constant ratio of AIBN, the grafting rate increases significantly with AA content; however, upon further increasing the AA content, the improvement in grafting efficiency slows down. The reason is that as the concentration of AA molecules increases, the contact opportunities between AA molecules and rubber molecules increase. Under the action of the initiator, free radical polymerization occurs to form a copolymer, and the grafting rate increases. However, since the number of reaction sites on rubber molecules is fixed, upon further increasing the monomer concentration, excess monomers undergo polymerization reactions, resulting in a downward trend in grafting efficiency. In addition, when the ratio of grafting monomer to rubber matrix is constant, as the concentration of initiator increases, the grafting rate and efficiency first rise rapidly and then gradually stabilize. During this process, the concentration of free radicals increases with the initiator content, as does the probability that the reaction sites can undergo the grafting reaction. Since the number of reaction sites is fixed, the grafting rate increases to a certain value after which it no longer increases significantly.

Setting the mass ratio of AA to NBR to 1 and the concentration of the initiator AIBN to 0.3% achieves the optimal grafting rate and efficiency.

#### 2.1.4. Synthesis of Sodium Polyacrylate Water-Absorbent Resin Gels

AA was dissolved in deionized water, and a 10% sodium hydroxide solution was added dropwise until the pH was neutral. Then, 2,2′-azobis[2-(2-imidazolin-2-yl) propane] dihydrochloride (AIBI) and glycerol were dissolved in the solution to obtain a mixed solution. ADSP and HPDSP of a certain mass ratio were added to deionized water, with a total mass of 20% of the deionized water. They were dissolved and pasted for a certain period of time at 80 °C and then cooled to room temperature to obtain a colloid liquid. The colloid liquid was added to the mixed solution, wherein the mass of the colloid liquid was 30% of that of the mixed solution. The mixture was sufficiently stirred and placed in a nitrogen atmosphere, followed by heating at 40 °C for 6 h to obtain the gel product. The product was dried under a vacuum pressure of 100 psi at 60 °C to obtain the sodium polyacrylate water-absorbent resin gel.

Modified starch contains a large number of hydrophilic hydroxyl groups in its molecular structure, and thus, grafting it with a water-absorbent resin gel significantly improves the water swelling ratio of the resin gel. Meanwhile, it also acts as a thickener and toughener to improve the resin gel’s resistance to shear damage. As a crosslinker, glycerol improves the crosslinking degree of the resin gel molecules, and the linear molecular chains can crosslink with each other to form a network, which enhances the strength and elasticity of the water-absorbent resin gel. With glycerol as the crosslinker, the crosslinking structure of sodium polyacrylate is as follows:


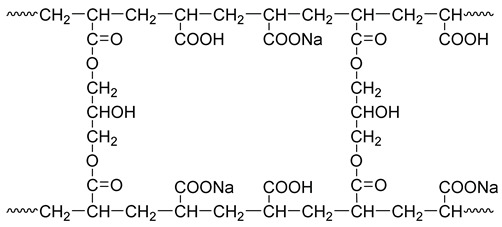


The water swelling ratio of sodium polyacrylate water-absorbent resin gel is affected by various factors, such as the monomer AA, crosslinker, and initiator. We used the orthogonal experimental method to determine the proportion of each component at the optimal water absorbency. The proportions of AA, crosslinker, initiator, and modified starch were set as factors for discussion, and a three-level orthogonal experimental design L9 (34) was applied for the study. The design of the orthogonal table and the results of the orthogonal experiments are presented in Table 1 and Table 2 below.

The range R reflects the significance of a factor to the result, with a larger R value indicating higher importance. A higher concentration of the reaction monomer AA results in a higher molecular weight of the resin gel. However, an excessively high concentration of AA causes the polymerization reaction to proceed too quickly, leading to a decrease in the molecular weight and the formation of crosslinked insoluble matter, affecting the final product. Modified starch contains a large number of hydrophilic groups, such as hydroxyl groups and, thus, can further improve the resin gel’s water absorption capacity. Glycerol acts as the crosslinker; when its concentration is too high, the crosslink density increases and inhibits the swelling of the crosslinked network, while when it is too low, the crosslink density decreases, resulting in a dissolution trend of the resin gel. Therefore, the concentration of glycerol should be reasonably controlled within a certain range. The initiator influences the polymerization reaction mainly via the concentration of generated free radicals. A free radical concentration that is too low leads to a decrease in the reaction rate of the initiated polymerization, while one that is too high leads to an excessively high crosslink density. This can easily cause explosive polymerization, leading to a decrease in the resin gel’s molecular weight and, thus, affecting its water absorbency. According to the data, in terms of their significance to the water-absorbent resin gel, the factors follow a descending order: AA concentration > ADSP/HPDSP > glycerol concentration > AIBI concentration. The optimal level design is 20% AA, an ADSP/HPDSP ratio of 2:1, 2% glycerol, and 0.5% AIBI.

#### 2.1.5. Thermal Stability Analysis

**Figure 5:**
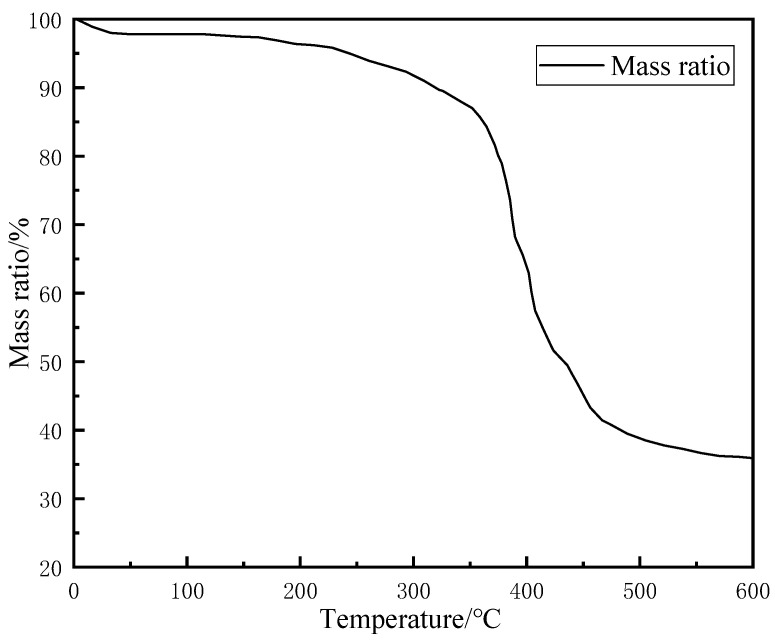
TG curve of resin-gel–rubber expandable LCM blend particles.

To study the thermal behavior of the resin-gel–rubber expandable LCM blend under high-temperature conditions, a TG experiment was carried out, and the results are shown in Figure 5.

As shown in Figure 5, the resin-gel–rubber expandable LCM blend showed a slight mass decrease of 2–5% when heated to 100 °C and a significant mass loss starting from 363 °C; from 470 °C, the mass decline gradually slows down. The curve stabilized from 560 °C and was approximately horizontal afterward, and the remaining mass was around 34%. Sodium polyacrylate has high hydrophilicity and easily absorbs water molecules in the air. Therefore, the mass loss at around 100 °C was mainly caused by the precipitation of water from the sodium polyacrylate water-absorbent resin gel mixed in the LCM. The LCM showed a significant downward trend starting from around 363 °C. At this stage, sodium polyacrylate and the rubber long-chain network molecular structure underwent heat damage, and the long molecular chains broke. Due to the grafting of sodium polyacrylate, the rubber’s polarity and crosslinking degree were increased. This resulted in larger intermolecular forces, inhibiting the intermolecular thermal motion to a certain extent and, thus, alleviating thermal decomposition. The final residue of around 34% was mainly rubber processing aids, such as zinc oxide and other ash residues. The results suggest the superior high-temperature resistance of the resin-gel–rubber expandable LCM blend.

### 2.2. Factors Influencing Water Absorption Capacity of Expandable LCM Blend

#### 2.2.1. Effect of Particle Size on Water Absorption Rate

**Figure 6:**
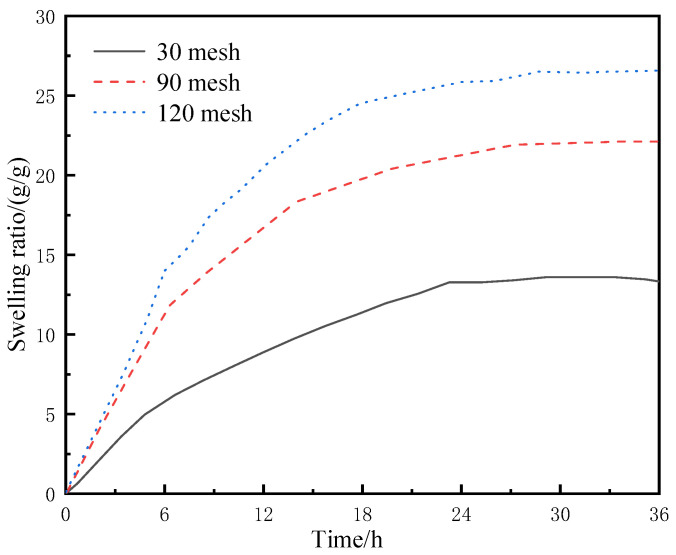
Swelling behavior of LCM particles of different particle sizes in deionized water.

The dried resin-gel–rubber expandable LCM blend was crushed with a pulverizer and screened with different meshes to obtain LCM particles of varied sizes. The particles were placed in separate flasks containing deionized water at 60 °C, and their water-swelling ratios were tested at regular time intervals. The experimental results are shown in Figure 6.

As shown in Figure 6, under the same conditions, LCM particles of larger mesh size (i.e., smaller particle size) had faster water absorption and swelling rates. The LCM particles of 120 mesh showed the fastest water absorption rate, and their mass was 25 times the original after water absorption for 12 h. In contrast, the mass of LCM particles of 30 mesh was 8 times the original after water absorption. This is because smaller particles have larger specific surface areas, which means they have a greater chance of contacting external water molecules and water molecules can migrate among water-absorbent resin-gel molecules in the LCM more easily; therefore, small LCM particles can absorb water and swell more quickly. However, the swelling stops at a certain point due to the equilibrium between the osmotic pressure difference and strain capacity of the particles. On the other hand, this is also because the water-absorbent resin gel is more likely to precipitate from the rubber in smaller particles.

#### 2.2.2. Effect of Temperature on Water Absorption Rate

**Figure 7:**
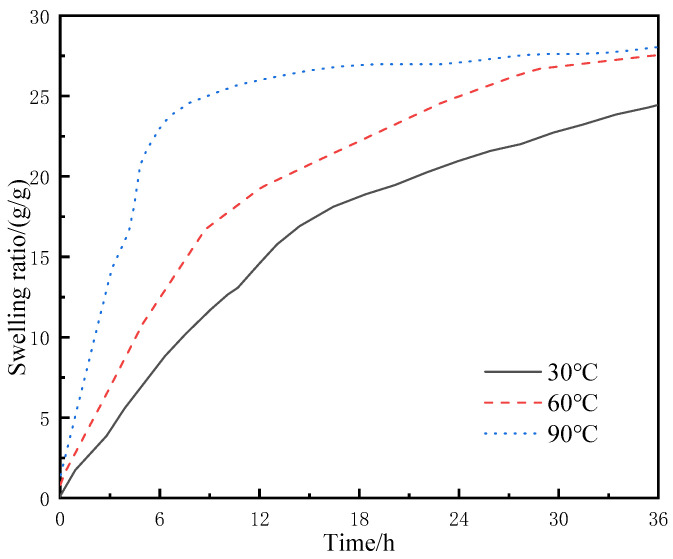
Swelling behavior of LCM particles under different temperatures in deionized water.

The resin-gel–rubber expandable LCM blend particles of 90 mesh were placed in flasks containing deionized water at 30, 60, and 90 °C, respectively. They were sampled at regular intervals to test the swelling ratios, and the experimental results are shown in Figure 7.

Compared with the water absorption rate and swelling ratio at 30 °C, higher temperatures resulted in faster water absorption of the LCM particles (i.e., higher swelling ratios were achieved in a shorter time at 60 and 90 °C). The swelling rate of LCM particles at 90 °C increased rapidly to 24% within 6 h and leveled off from 24% to 28% during the 6th to 36th hour of 90 °C swelling. The swelling ratios of LCM particles of the same size were relatively close at 36 h, with the mass reaching over 24 times the original mass; however, at 6 h, the mass of the LCM particles under 30 °C reached about 7 times the original mass, while those under 60 and 90 °C reached about 12 and 22 times the original mass, respectively. There are two reasons behind this. First, with the rise in external temperature, the thermal motion of the resin-gel molecules became more intense, and the molecular chain segments and side groups entered active thermal motion states. Thus, the temperature rise increased the energy of molecular thermal motion, and when the energy of motion units overcame the minimum energy limit of a certain state, the motion unit entered the active state. Second, as the temperature rose, the molecular spacing increased [20,21]. Thus, the water molecules entered the internal molecular space of the resin gel more easily under the action of osmotic pressure and the capillary effect, achieving a higher swelling ratio more quickly.

#### 2.2.3. Effect of Salinity on Swelling Ratio

Salinity tolerance is an important indicator for LCMs. The concentration, strength, and valence of cations in the ambient liquid are important factors affecting the water absorption capacity of hydrophilic polymer LCMs. When cations are present in the electrolyte solution, they interact with the weakly electrolytic water, leading to a significant decrease in the activity of water molecules. The decrease in the charge concentration difference then leads to a decrease in osmotic pressure, which, in turn, further lowers the hydrophilic polymers’ ability to absorb water.

Omidian and Hashemi et al. [22] proposed the following expression for the relationship between the ion concentration in ambient liquid and the water absorption capacity of a polymer:

(3) $$V=K{\left(\frac{1}{C}\right)}^{n},$$

where C denotes the ion concentration in the ambient liquid, K and n denote the property parameters of the polymer, which are independent of the external environment, and V denotes the water absorption capacity of the polymer, which, according to the theory, decreases with the ion concentration in the environment.

**Figure 8:**
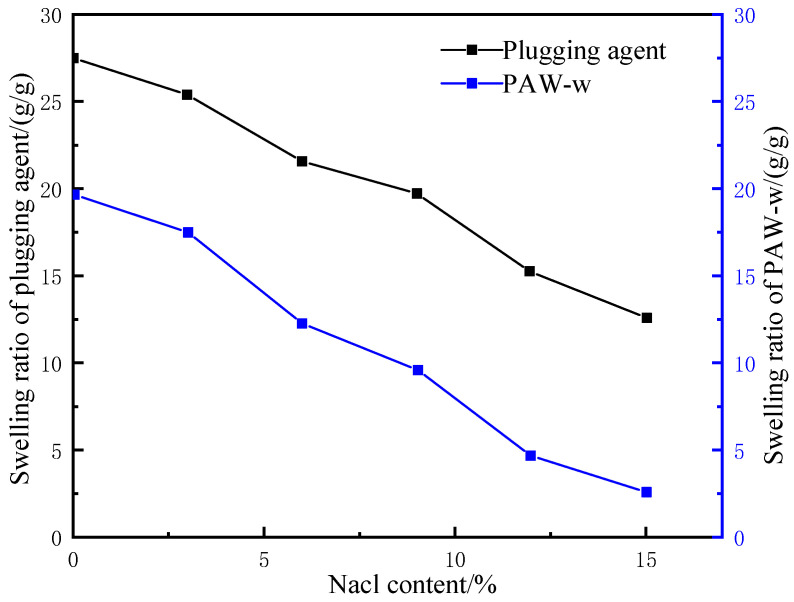
Water absorption and swelling test of LCM under different brine concentrations.

When encountering brine formation during drilling, the LCM can react with the brine and cause plugging failure. To avoid this scenario, it is important to test the salinity tolerance of LCMs. For this purpose, brines with concentration gradients of 3%, 6%, 9%, 12%, and 15% NaCl at 60 °C were prepared. The corresponding water absorption properties of the LCM in brines were observed and compared with that in deionized water. The resin-gel–rubber expandable LCM blend particles of 90 mesh were used for the water absorption test, with a total of 36 h of immersion. To evaluate the property more directly, the homemade polyacrylamide water-absorbent resin gel PAM-w was used as a comparison and tested under the same experimental conditions. The experimental results are presented in Figure 8.

According to Figure 8, the swelling ratio decreased with increased brine concentration for both the LCM and PAM-w. In deionized water, the mass of the LCM and PAM-w after water absorption reached 27 and 20 times the original, but the corresponding factors decreased to 17 and 8, respectively, in 10% brine. However, in comparison, the LCM maintained an overall higher water absorption capacity than PAM-w. This is because the molecular chain length of the crosslinker affected the mesh size in the water-absorbent resin-gel network structure, thus, influencing the salinity tolerance. On the one hand, as a polymer crosslinker, glycerol has a longer molecular chain than traditional small molecule crosslinkers, which improved the binding power of the resin-gel network to water molecules, maintaining a relatively high water absorbency in brine of the same concentration. On the other hand, glycerol performs bridge crosslinking via dehydration and ester formation with sodium polyacrylate, introducing non-ionic ester groups and hydroxyl groups into the anionic polymer molecular chain. Bridge crosslinking reduced the resin gel’s water solubility, which, in turn, improved its salinity tolerance.

### 2.3. Lost Circulation Control Performance of Expandable LCM Blend

A plugging material tester QD-2 was used to determine the lost circulation control performance of the expandable LCM blend, and the specifications of the fracture plates were 1 and 2 mm. The LCM slurries used for the experiments were prepared according to the conditions listed in Table 3, with each slurry being 4000 mL. A nitrogen source of 12 MPa was used as the pressure source, and the experimental pressure was 0–7 MPa. First, nitrogen was connected, and the pressure was maintained at 1.0 MPa for 10 min to record the total fluid loss volume. Then, the pressure was increased by 1.5 MPa each time, and the target pressure was maintained for 10 min to record the cumulative fluid loss volume of each LCM slurry. The results are shown in Table 4.

To ensure the same experimental conditions, the LCM slurries of all formulations were pretreated in a water bath at 60 °C for 8 h and stirred again before use. The experiments employing formulations 1 and 3 were performed with a 1-mm fracture plate, while those employing formulations 2 and 4 were performed with a 2-mm fracture plate. The experimental results are recorded in Table 4.

The results show that the use of the resin-gel–rubber expandable LCM blend significantly reduced the LCM slurry’s fluid loss volume, showing a bearing resistance capacity of 7 MPa. This indicates that the resin-gel–rubber expandable LCM blend particles had strong toughness and elastic deformation ability after absorbing water and could squeeze into the gaps in rigid particles through deformation; therefore, the plugging was more dense and compact, achieving superior lost circulation control effects.

### 2.4. Fracture Lost Circulation Control Mechanism of Expandable LCM Blend

**Figure 9:**
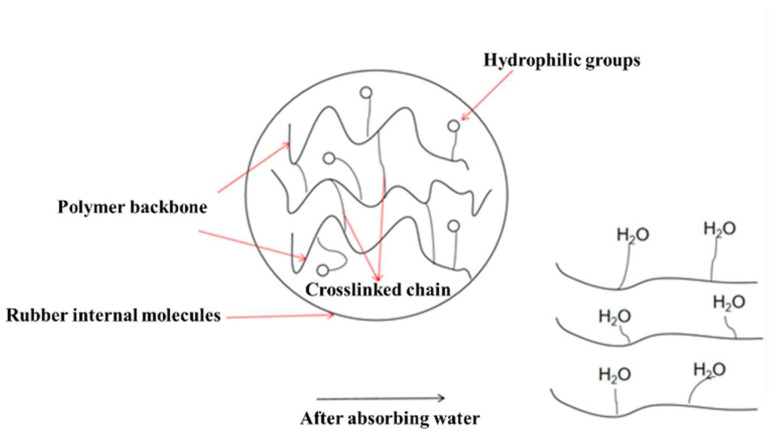
Water absorption mechanism of resin-gel–rubber expandable LCM blend.

In this study, we prepared an expandable LCM blend by blending a rubber matrix with a water-absorbent resin gel, and the molecules of the compatibilizer used contained hydrophilic groups at one end and lipophilic groups at the other end, which reduced the interfacial energy and improved the compatibility between the rubber and the resin gel (Figure 9). When contacting water, the water molecules entered the water-absorbent resin gel through capillary action and surface adsorption or diffusion and combined with the hydrophilic groups, such as hydroxyl. Due to the pressure difference between the interior and exterior, water absorption and swelling occurred macroscopically, leading to deformation of the LCM. When the rubber’s resistance to deformation was balanced with the osmotic pressure, the maximum swelling ratio was reached [23,24].

**Figure 10:**
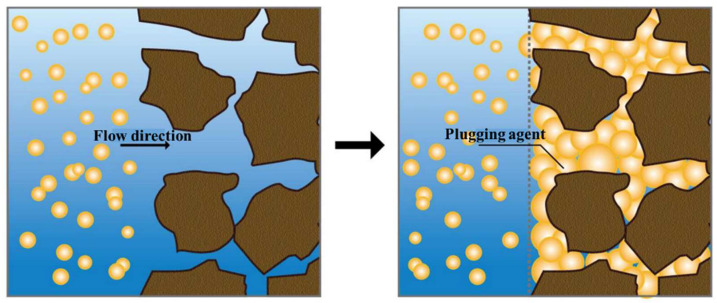
Schematic diagram of the mechanism of water absorption and swelling for lost circulation control in fractures.

When entering the lost circulation channels in the formation, the LCM particles that were less affected by gravity could reach the deep part of the small fractures. Under the effects of temperature and osmotic pressure, the LCM particles started to absorb water and swell, and they continuously extruded and deformed under the liquid column pressure (Figure 10). The particles piled up and filled the fractures, forming a dense “plugging plug”. Therefore, the internal and external pressures were balanced to ensure that lost circulation was minimized during drilling. Meanwhile, with its high toughness and strength, the dense plugging layer formed could also withstand some pressure fluctuations caused by the drawdown of the drill pipe.

## 3. Conclusions

1. The developed resin-gel–rubber expandable LCM blend was mainly composed of sodium polyacrylate resin gel and NBR. On the basis of mechanical blending, the use of the homemade amphiphilic compatibilizer reduced the interfacial tension between the two phases, which greatly improved the compatibility of the resin gel in the rubber matrix. The ratio of the resin gel, rubber, and compatibilizer was 0.25:1:0.2.
2. The resin-gel–rubber expandable LCM blend absorbed water and swelled under the action of the water-absorbent resin gel. Under certain conditions, smaller particle size and higher temperature were associated with faster water absorption and swelling rates of the LCM particles. Meanwhile, the salinity degree affected the mesh size in the network structure of the resin gel and, thus, influenced the swelling ratio of the LCM particles.
3. The resin-gel–rubber expandable LCM blend particles had excellent toughness and elastic deformation ability after absorbing water. They could swell, squeeze, deform, and fill the fractures, achieving a bearing resistance capacity of 7 MPa for 2-mm fractures and displaying superior lost circulation control performance for fractures.

## 4. Materials and Methods

### 4.1. Materials

The materials included the following: methacrylic acid (A.R. grade, Wuxi Yatai United Chemical Co., Ltd., Wuxi, China), sodium hydroxide (A.R. grade, Wuxi Yatai United Chemical Co., Ltd.), sodium persulfate (A.R. grade, Wuxi Yatai United Chemical Co., Ltd.), 2,2′-[azobis(1-methylethylidene)]bis [4,5-dihydro-1H-imidazole dihydrochloride (industrial grade, Shanghai Juqi Chemical Technology Co., Ltd., Shanghai, China), acetylated distarch phosphate ((ADSP) industrial grade, Zhucheng Depu Trade Co., Ltd., Zhuhai, China), hydroxypropyl distarch phosphate ((HPDSP) industrial grade, Nanjing Songguan Biotechnology Co., Ltd., Nanjing, China), deionized water (made in the laboratory), powder nitrile rubber ((NBR) industrial grade, Beijing Yuda Xing Industry and Trade Co., Ltd., Beijing, China), polymerized sulfur ((insoluble sulfur) industrial grade, Guangzhou Liben Rubber Material Trade Co., Ltd., Guangzhou, China), zinc oxide (Guangzhou Liben Rubber Material Trade Co., Ltd.), stearic acid 1801 (Guangzhou Liben Rubber Material Trade Co., Ltd.), toluene (A.R. grade, Yixing Kaiteng Chemical Co., Ltd., Yixing, China), acetone (industrial grade, Shandong Fengcang Chemical Co., Ltd., Zibo, China), antioxidant RD (Shanghai Panren International Trade Co., Ltd., Shanghai, China), accelerator NA-22 (Shanghai Panren International Trade Co., Ltd.).

### 4.2. Equipment

The equipment included the following: double-roller open mill YX-230 (Dongguan Yunxin Machinery Manufacturing Co., Ltd., Dongguan, China), closed mixer (Wuxi Erxiang Machinery Co., Ltd., Wuxi, China), double-layer flat vulcanizer ZG-10T (Dongguan Zhenggong Electromechanical Equipment Technology Co., Ltd., Dongguan, China), ultrasonic disperser JP-020 (Shenzhen Jiemeng Cleaning Co., Ltd., Shenzhen, China), vacuum drying oven BPZ-6120-2B (Shanghai Yiheng Scientific Instrument Co., Ltd., Shanghai, China), electronic balance JA1003 (Shanghai Fangrui Instrument Co., Ltd., Shanghai, China), thermogravimetric (TG) analyzer TGA400 (imported from the United States through Nanning Linao Instrument Co., Ltd., Nanning, China), German Zeiss field emission scanning electron microscope SIGMA500 (Beijing Opton Optical Technology Co., Ltd., Beijing, China), tensile tester YHS-216W-200N-7 (Shanghai Yihuan Instrument Technology Co., Ltd., Shanghai, China), plugging material tester QD-2 (Qingdao Haitongda Special Instrument Co., Ltd., Qingdao, China), punching machine CPJ-30 (Chengde Kecheng Testing Machine Co., Ltd., Chengde, China).

### 4.3. Evaluation Methods

#### 4.3.1. Water Swelling Ratio

A certain number of resin-gel–rubber expandable LCM blend particles were taken as the sample and weighed accurately using an electronic balance (accuracy 0.01 g) to record the initial mass M_0_. The sample was then soaked in deionized water at 60 °C for 24 h. Upon removing the sample from the water, the surface moisture was quickly absorbed using absorbent paper towels, and the mass was weighed as Mn. The water-swelling ratio (%) was calculated as the percentage change in mass after water absorption:

(4) $$W_{n}=\frac{M_{n}-M_{0}}{M_{0}}\times 100\%,$$

#### 4.3.2. Tensile Strength Test

After the final vulcanization, the sample was left at room temperature for 24 h, and two sample strips with a thickness of 3 mm, a length of 20 mm, and a width of 10 mm were prepared from the resin-gel–rubber expandable LCM blend using a CPJ-30 punching machine. A tensile tester was used to determine the tensile strength of the samples at a stretching rate of 40 mm/min. The tensile strength σ (MPa) was calculated using the following formula:

(5) σ=F(b·h),

where F is the tensile load, and b and h are the width and thickness of the sample strip, respectively.

#### 4.3.3. Lost Circulation Control Performance

The QD-2 plugging material tester was used to determine the lost circulation control performance. First, the tester’s screw cap was unscrewed, and the slurry cylinder and three-way valve were removed. A fracture plate of a certain size was then screwed into the outlet of the ball valve and tightened. Next, a 4000-mL measuring cylinder was placed at the outlet of the ball valve, and the prepared LCM slurry was stirred thoroughly and poured into the measuring cylinder, which was then sealed. Afterward, the nitrogen source was connected, and the bleed valve of the three-way component was closed to ensure airtightness. The connecting valve was then opened to introduce nitrogen for pressurization, and a stable pressure was maintained at 100 psi. Finally, the total volume of liquid lost within 10 min after opening the ball valve was recorded.

#### 4.3.4. TG Test

TG analysis was employed to test the gel particles’ thermal stability using a TGA400 TG analyzer (METTLER TOLEDO, Columbus, OH, USA). The sample was heated from 30 °C to 600 °C at a heating rate of 10 °C/min under a protective nitrogen flow of 100 mL/min. The change in the total mass of the gel with temperature was recorded, and a TG curve was plotted.

## Figures and Tables

**Table 1:** Orthogonal conditions for the synthesis of sodium polyacrylate resin gels.

Level	AA Concentration/%	Glycerol Concentration/%	AIBI Concentration/%	ADSP/HPDSP
1	15	2	0.3	1:1
2	20	3	0.5	1:2
3	25	4	0.7	2:1

**Table 2:** Orthogonal experimental results for the synthesis of sodium polyacrylate resin gels.

Experiment No.	Factor Level				Swelling Ratio
	AA Concentration/%	Glycerol Concentration/%	AIBI Concentration/%	ADSP/ HPDSP	
1	1	1	1	1	36.83
1	1	2	2	2	46.32
1	1	3	3	3	47.62
2	2	1	2	3	62.30
2	2	2	3	1	37.69
2	2	3	1	2	59.26
3	3	1	3	2	42.65
3	3	2	1	3	26.48
3	3	3	2	1	27.66
K1	130.77	141.78	122.57	102.18	-
K2	159.25	110.49	136.28	148.23	-
K3	96.79	134.54	127.96	136.40	-
k1	43.59	47.26	40.86	34.06	-
k2	53.08	36.83	45.43	49.41	-
k3	32.26	43.85	42.65	45.47	-
R	20.82	10.43	4.57	15.35	-
Optimal Conditions	20	2	0.5	2:1	-

**Table 3:** Formulations of LCM slurries for comparison experiments.

Formulation No.	Formulation
1	4% bentonite + 2% SQD-98 (fine) + 2% limestone (40–80 mesh) + 6% FDL-1 (80–120 mesh)
2	4% bentonite + 2% SQD-98 (medium) + 2% limestone (20–40 mesh) + 6% FDL-1 (40–80 mesh)
3	4% bentonite + 2% SQD-98 (fine) + 2% limestone (40–80 mesh) + 6% resin-gel–rubber expandable LCM blend (80–120 mesh)
4	4% bentonite + 2% SQD-98 (medium) + 2% limestone (20–40 mesh) + 6% resin-gel–rubber expandable LCM blend (40–80 mesh)

**Table 4:** Total fluid loss volume of each LCM slurry during pressurization.

Pressure /MPa	Cumulative Fluid Loss Volume/mL			
	Formulation 1	Formulation 2	Formulation 3	Formulation 4
1.0	224	337	65	154
2.5	427	458	125	234
4.0	689	796	363	406
5.5	1064	Total loss	365	406
7.0	Total loss	Total loss	365	406

## Data Availability

The data are contained within the article.
